# A reproducible pretreatment mucosal-inflammatory-remodeling state is associated with induction-phase anti-tumor necrosis factor non-response in ulcerative colitis

**DOI:** 10.3389/fgene.2026.1845510

**Published:** 2026-07-30

**Authors:** Hongwei Zheng, Xin Zhuang, Wenbiao Chen

**Affiliations:** 1 Quanzhou Medical College, Quanzhou, Fujian, China; 2 Quanzhou First Hospital Affiliated to Fujian Medical University, Quanzhou, Fujian, China

**Keywords:** anti-tumor necrosis factor, epithelial remodeling, infliximab, mucosal transcriptome, myeloid cells, primary non-response, single-cell RNA-sequencing, ulcerative colitis

## Abstract

**Background:**

Primary non-response to anti-tumor necrosis factor (anti-TNF) therapy remains a major clinical challenge in ulcerative colitis (UC); however, public-data transcriptomic studies often yield unstable single-gene biomarkers and limited replication.

**Methods:**

We analyzed three independent pretreatment UC mucosal transcriptome cohorts from infliximab-treated patients (total n = 79). The induction response was assessed at 4–8 weeks according to the original definition in each study. To prioritize cross-cohort biology over single-probe overlap, we integrated recurrent effect direction, pathway convergence, and sample-level scoring. We defined a 68-gene consensus signature for interpretation, evaluated generalization with leave-one-dataset-out (LODO) scoring, tested attenuation after adjusting for baseline inflammatory/remodeling proxies, and examined cellular localizations in public epithelial (GSE116222) and rectal immune (GSE125527) single-cell references.

**Results:**

The 68-gene consensus signature was higher in non-responders across all three cohorts. In the LODO analysis, the held-out non-response axis remained elevated in the held-out non-responders, with a pooled random-effects size of Hedges’ g = 1.36 (95% confidence interval: 0.83‐1.89; *p* = 5.65 × 10^−7^; I^2^ = 0%). The pathway analyses consistently implicated inflammatory responses, TNF-α/NF-κB, IL-6–JAK–STAT3, interferon signaling, hypoxia, and epithelial–mesenchymal transition (EMT), whereas oxidative phosphorylation was enriched in the responders. Adjustments for inflammatory proxies with and without EMT attenuated the effects, but the associations remained directionally positive in all cohorts and retained nominal significance in two cohorts under the strictest model. In public single-cell references, the signature activities were enriched in cycling/stem-like and stress-inflammatory epithelial states and were highest in the rectal myeloid/dendritic cells (M/DCs).

**Conclusion:**

In the infliximab-treated UC cohorts, induction-phase non-response is associated with reproducible pretreatment mucosal-inflammatory-remodeling states that remain evident in the held-out cross-cohort analyses while localizing most strongly to rectal M/DCs and stress-associated epithelial states in public single-cell references. The signal overlaps substantially with the baseline inflammatory/remodeling burden, but its direction is consistent across the cohorts. These findings support a biologically coherent disease-state framework rather than a deployable clinical predictor.

## Introduction

Ulcerative colitis (UC) is a chronic inflammatory disorder of the colonic mucosa and is marked by relapsing tissue injury, persistent immune activation, and substantial therapeutic heterogeneity. Although anti-tumor necrosis factor (anti-TNF) antibodies remain an important treatment option for moderate-to-severe UC, a substantial fraction of patients show primary non-response despite clinical assessment before treatment. This clinical problem prolongs exposure to ineffective therapy, delays alternative treatment, and may reflect a distinct pretreatment biological state rather than random therapeutic failure (Rutgeerts et al., 2005; Raine et al., 2022; Moran et al., 2025; Vieujean et al., 2025). Modern management approaches for UC increasingly emphasize treat-to-target strategies and endoscopic/histologic endpoints, but a therapeutic ceiling persists even with advanced therapies (Turner et al., 2021; Dignass et al., 2023; West et al., 2023; Ma et al., 2024). Therefore, primary non-response remains common and continues to drive biomarker discovery (Leone et al., 2023; Anjie et al., 2023). The main goal of these efforts is to define pretreatment biological states that help identify patients who are less likely to benefit from TNF neutralization alone (Gisbert and Chaparro, 2020; Cui et al., 2021; Cui et al., 2022; Chen et al., 2025).

Many transcriptomic studies have attempted to identify the pretreatment biomarkers of anti-TNF response in UC. Early bulk mucosal studies proposed multigene predictors, but their cross-cohort stability was variable (Arijs et al., 2009a; Arijs et al., 2009b; Toedter et al., 2011). Oncostatin M (OSM)-centered inflammatory signaling subsequently emerged as a prominent correlate of anti-TNF non-response in inflammatory bowel disease (IBD), supporting a biologically plausible myeloid/stromal inflammatory model of therapeutic failure (West et al., 2017). Similarly, later studies implicated immune activation and trafficking programs (Belarif et al., 2019; Yang et al., 2023; Peña-Cearra et al., 2023; Pu et al., 2024). More recently, omics-driven and network or machine-learning approaches have not only expanded the candidate space but also underscored biomarker instability when the sample size is modest relative to the feature dimensionality (Ou et al., 2025; Wang et al., 2025a; Shanthamallu et al., 2024; Varma and Simon, 2006).

Public-data transcriptomic studies are limited by practical challenges, such as recovering biologically coherent signals across cohorts without turning cohort reuse into circular validation (Varma and Simon, 2006). We addressed this by emphasizing the recurrent effect direction, pathway convergence, and held-out effect-size estimation. We therefore considered three linked questions: can pretreatment primary anti-TNF non-response in UC be captured as a coherent mucosal program across independent cohorts; does this program localize to plausible cellular compartments in public single-cell references; and does the association persist after accounting for baseline inflammatory/remodeling burden? These questions are most relevant in a disease-biology framework, where the aim is to characterize the pretreatment tissue state. To address these questions, we performed a hypothesis-driven analysis of three pretreatment UC mucosal transcriptome cohorts of infliximab-treated patients. Additionally, we defined a compact 68-gene consensus signature for interpretation, evaluated generalization with leave-one-dataset-out (LODO) scoring, examined attenuation after progressively stricter inflammatory/remodeling adjustments, and used public epithelial and immune single-cell references to provide the cellular context. Thus, we sought to describe the pretreatment biology of anti-TNF non-response in UC such that it is reproducible and biologically coherent.

## Methods

### Study design

We conducted a multicohort public-data transcriptomic study focused on pretreatment primary anti-TNF non-response in UC. Here, three independent infliximab-treated UC mucosal transcriptome cohorts were analyzed. We used a fixed 68-gene consensus signature for interpretation and a separate LODO scoring scheme for held-out effect-size estimation. Public single-cell references were used only to provide the cellular context and were not treated as discovery cohorts.

### Public bulk transcriptome cohorts

Three cohorts were used from the Gene Expression Omnibus (GEO) database for the core analysis: GSE12251, GSE16879, and GSE23597. Of these, GSE12251 served as the discovery cohort for pathway prioritization, while GSE16879 was used for directional replication, and GSE23597 was considered as an additional independent cohort. For the LODO evaluation, each cohort was treated as the held-out dataset in turn. For the primary analysis, we retained only the pretreatment UC mucosal biopsies and harmonized the original study outcomes to a binary responder/non-responder framework while preserving the endpoint definition and assessment timepoint of each cohort.

In GSE12251, all analyzed samples were pretreatment colonic biopsies obtained at week 0; the study annotation reflected week-8 endoscopic and histologic healing, so the primary non-response was defined as the absence of this combined healing endpoint. In GSE16879, the biopsies were obtained before treatment and again at 4–6 weeks after the first infliximab infusion, with the responses being classified from the endoscopic and histologic findings at the 4–6-week assessment timepoint; we retained the pretreatment UC biopsies and used the responder/non-responder classification of this study for the binary outcome. In GSE23597 (ACT 1 sub-study), the biopsies were collected at baseline and during follow-up; we retained the pretreatment biopsies from the infliximab-treated arms and used the study’s week-8 response classification to define induction response versus non-response.

Because the outcome definitions were not identical across the three cohorts (4–6 weeks vs. 8 weeks, with one cohort using an endoscopic-plus-histologic healing endpoint), we treated the harmonized outcome as a pragmatic induction (4–8-week) non-response definition. The biologic-naive status and exact biopsy locations were not consistently extractable from the deposited sample annotations across all three cohorts, so these factors were not used for stratified modeling. Therefore, the main cross-cohort evidence is summarized using held-out LODO evaluation and random-effects pooling rather than any single-cohort estimate.

### Bulk expression preprocessing

Normalized expression matrices were obtained from the GEO series-matrix files and organized into phenotype tables and expression matrices restricted to the selected core samples (Edgar et al., 2002). Each cohort was processed independently to preserve the cohort-native normalization while avoiding naive cross-platform pooling. The probe-level matrices were analyzed first and then mapped to gene symbols for cross-cohort directionality summaries. The replication standards emphasized effect direction and pathway/signature consistency rather than pooled expression magnitude.

### Differential expression analysis

Within each bulk cohort, the responder versus non-responder comparisons were performed using limma (Ritchie et al., 2015). The linear models used response status as the main term, and the moderated t-statistic from the responder versus non-responder contrast was retained for downstream ranking. The probe-level differential results were mapped to gene symbols using platform annotation rules for the Affymetrix GPL570 platform; when multiple probes were mapped to the same gene symbol, a single representative gene-level record was retained for the cross-cohort direction summary and scoring.

### Pathway analysis

Hallmark pathway analysis was performed on the gene-level limma moderated t-statistics using a preranked gene-set enrichment approach (Subramanian et al., 2005; Liberzon et al., 2015; Korotkevich et al., 2016). The ranking statistic was the responder versus non-responder moderated t-value; therefore, negative normalized enrichment scores (NESs) in the raw enrichment outputs corresponded to pathways elevated in the non-responders, whereas positive NESs corresponded to pathways elevated in the responders. For figure readability, the signed NES values were reoriented so that the positive values indicate non-responder enrichment. Hallmark gene sets were obtained from the MsigDB database, and enrichment was performed using the fgsea package. The pathways were summarized per cohort and compared across cohorts using direction consistency, false discovery rate (FDR), and nominal significance counts across the datasets. The analyses were performed using R software version 4.4.3 with limma 3.62.2, fgsea 1.32.0, msigdbr 25.1.1, and Python 3.13.7.

### Consensus signature and LODO evaluation

We used two related signatures for different purposes. The fixed 68-gene consensus signature was not selected from prioritized pathways; it was defined strictly from the bulk differential expression results as genes that were directionally elevated in the non-responders and nominally significant at *p* < 0.01 in all three cohorts. This fixed signature was used for biological interpretation, attenuation analyses, and single-cell localization. The generalization was assessed separately with LODO scoring, in which each cohort was evaluated with a gene set derived only from the other two cohorts (directionally elevated in non-responders and nominal *p* < 0.05 in both training cohorts). Therefore, the held-out LODO gene sets differed from the fixed consensus signature by design and were used for held-out effect-size estimation rather than definition of the interpretable 68-gene set. For interpretability, we also annotated the 68 consensus genes into broad descriptive groups based on known gene functions and annotations: immune/myeloid activation and antigen-presentation markers, cytokine/inflammatory and stress-signaling mediators, tissue-remodeling/stromal/endothelial or epithelial-stress genes, and intracellular regulatory/stress-response genes. These groups were used only to summarize the signature composition shown in Supplementary Table S1; furthermore, we did not treated as evidence that any subset is independent of inflammation.

### Effect-size and replication analyses

Module scores were calculated within each cohort from the genes available on the corresponding microarray platforms. For each signature gene, one representative probe was retained to standardize the expression across the pretreatment samples within that cohort. The sample-level module score was the unweighted mean of these within-cohort gene z-scores, with all included genes being oriented so that a higher expression corresponded to the non-responder-enriched direction. Thus, the scores are relative within-cohort quantities rather than absolute expression measurements. Standardized effect sizes were calculated for the non-responders versus responders using Hedges’ g with confidence intervals (CIs). The primary replication evidence was summarized using the LODO axis (held-out evaluation) with fixed-effects and random-effects meta-style summaries, and the heterogeneity was summarized using I^2^ (Higgins and Thompson, 2002). The consensus-signature effect sizes were treated as descriptive in-sample upper bounds.

### Specificity analysis against baseline inflammatory burden

We next evaluated whether the consensus signature largely reflected the baseline inflammatory and remodeling burden. First, we created a simple inflammatory proxy using the within-cohort z-scored expressions of seven recurrent mucosal inflammatory genes observed in the non-responders (*IL1B*, *TLR2*, *TNFAIP6*, *NAMPT*, *PTGS2*, *CXCR2*, and *TREM1*) using a single representative probe per gene when available. These genes were selected because they were repeatedly elevated in the non-responders in the present cross-cohort analyses and are consistent with the mucosal inflammatory biology discussed in prior anti-TNF and IBD biomarker studies (Cui et al., 2021; Arijs et al., 2009a; Arijs et al., 2009b; Toedter et al., 2011; West et al., 2017). We then constructed stricter Hallmark-based inflammatory proxies with and without epithelial–mesenchymal transition (EMT) from the available genes in the corresponding MSigDB gene sets. Because these proxies overlap biologically with the consensus signature, the stricter models were interpreted as sensitivity analyses rather than formal causal disentanglement. Within each cohort, the linear models estimated the associations between the consensus-signature score and response status before and after adjustment for these proxies.

### Class-level anti-TNF sensitivity analysis (golimumab cohort)

As a sensitivity analysis beyond infliximab, we evaluated the fixed 68-gene consensus signature in a golimumab-treated UC cohort (GSE92415) derived from the PURSUIT-SC induction study (Sandborn et al., 2014; Telesco et al., 2018). Here, we retained the pretreatment mucosal biopsies and used the study’s week-6 response classification to define induction response versus non-response. This analysis was treated as supportive class-level evidence and was not incorporated into the core three-cohort effect-size pooling.

### Single-cell localization support (processed public references)

The single-cell analyses involved processed public data and were intended to provide cellular context rather than discovery evidence.

#### Epithelial reference

The GSE116222 dataset served as the epithelial reference and corresponds to the single-cell atlas of human colon in UC (Smillie et al., 2019). Using the processed expression matrix and accompanying metadata, we assigned broad epithelial states using a curated marker panel and a simple maximum marker-score rule. The 68-gene consensus signature was then scored at the single-cell level using the genes available in the reference matrix (Smillie et al., 2019; Parikh et al., 2019). Inter-state score differences were assessed using the Kruskal–Wallis test and one-versus-rest Wilcoxon rank-sum tests with Benjamini–Hochberg adjustment.

#### Immune rectal reference

The GSE125527 dataset served as the immune reference. We used the processed expression matrix and metadata tables, restricted the analysis to rectal tissue cells, and summarized the signature activity across the annotated immune cell classes using genes present in the reference matrix (Parikh et al., 2019). Inter-cell-class differences were assessed separately for the diseased and healthy rectal cells using the Kruskal–Wallis test and one-versus-rest Wilcoxon rank-sum tests with Benjamini–Hochberg adjustment. These tests were used as descriptive localization support at the cell level but not as donor-level inferential tests. The reported single-cell summary values represent within-reference relative activities and should be interpreted as enrichment patterns rather than direct evidence of cell-of-origin.

## Results

### Cohort assembly and analysis design

The three pretreatment UC mucosal transcriptome cohorts from infliximab-treated patients were assembled in a discovery-replication framework. The analysis set included 23 patients from GSE12251 (12 responders, 11 non-responders), 24 from GSE16879 (8 responders, 16 non-responders), and 32 from GSE23597 (25 responders, 7 non-responders), for a total of 79 patients, as described in Table 1.

**TABLE 1:** Summary of the core cohorts used for the primary claim.

Cohort	Role	Therapy	Platform	Endpoint/timepoint (induction)	Biopsy/source notes from deposited records	n	Responders	Non-responders
GSE12251	Discovery	Infliximab	GPL570	Week-8 endoscopic-plus-histologic healing; non-response = absence of combined healing	Pretreatment colonic biopsies at week 0	23	12	11
GSE16879	Replication	Infliximab	GPL570	4–6-week endoscopic and histologic response after first infusion	Pretreatment mucosal biopsies from actively inflamed UC mucosa	24	8	16
GSE23597	Cohort 3	Infliximab	GPL570	Week-8 response classification in the ACT 1 gene-expression sub-study	Pretreatment colonic biopsy samples from infliximab-treated arms	32	25	7

Endpoint definitions and biopsy/source notes are reported from the deposited GEO records and associated study descriptions. Biologic-naive status and granular anatomic biopsy locations were not consistently available across all three cohorts and were therefore not modeled.

**FIGURE 1:**
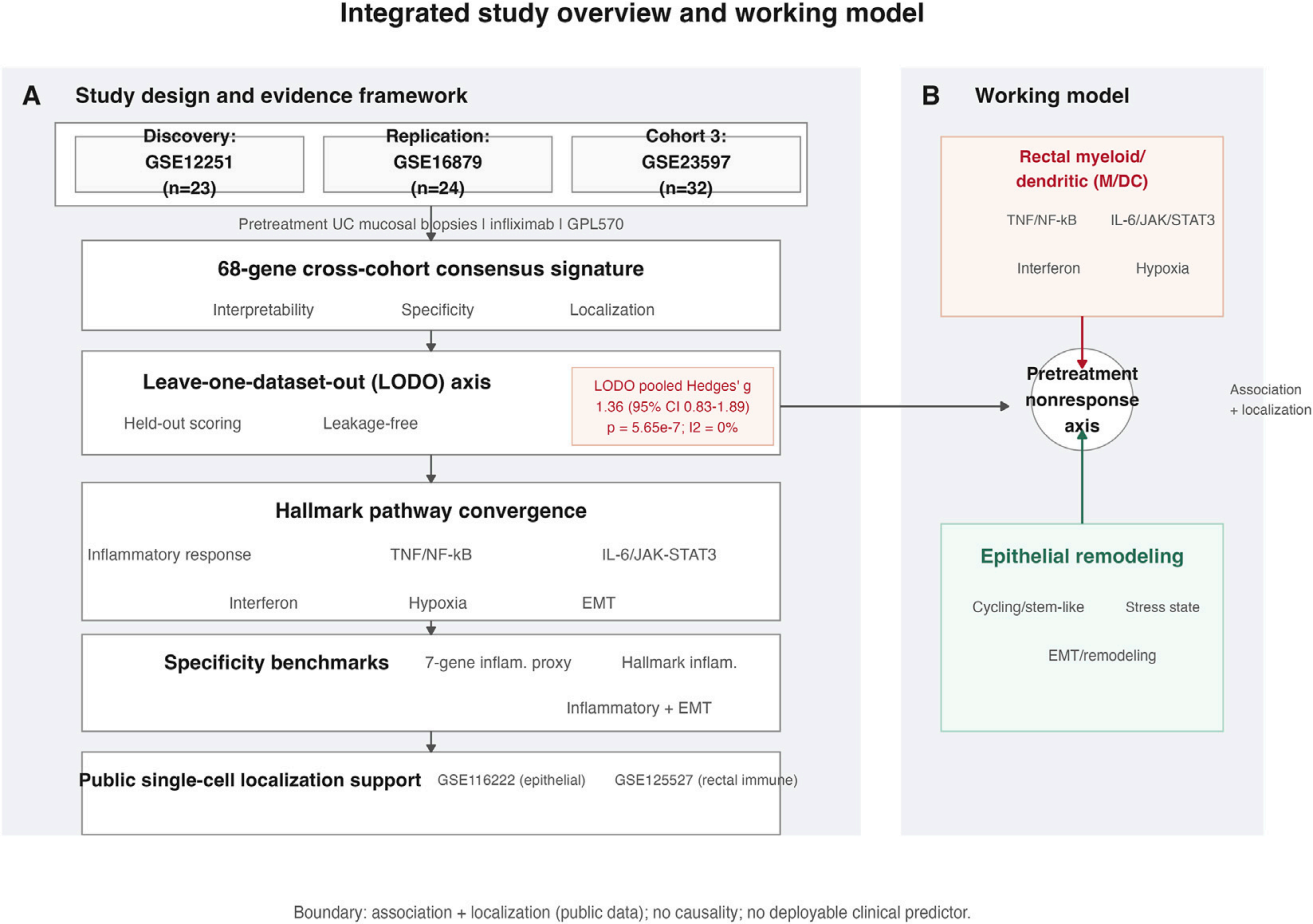
Overview of the study design, evidence framework, and working model for pretreatment anti-tumor necrosis factor (anti-TNF) non-response in ulcerative colitis (UC). **(A)** Study design and evidence framework showing the three bulk cohorts (GSE12251, GSE16879, and GSE23597), the fixed 68-gene consensus signature used for interpretation and localization, leave-one-dataset-out (LODO) analyses used for held-out effect-size estimation (pooled random-effects Hedges’ g = 1.36, 95% confidence interval: 0.83-1.89; p = 5.65 x 10^−7^; I^2^ = 0%), attenuation analyses relative to inflammation with and without epithelial-mesenchymal transition (EMT) proxies, and cellular contexts from epithelial (GSE116222) and rectal immune (GSE125527) single-cell references. **(B)** Working model summarizing how a pretreatment inflammatory-remodeling axis may link myeloid/dendritic-cell activation with epithelial stress/remodeling in anti-TNF non-response.

The response assessment definitions and timepoints were cohort-native: GSE12251 used week-8 endoscopic-plus-histologic healing; GSE16879 used endoscopic and histologic responses at 4–6 weeks after first infliximab infusion; GSE23597 used week-8 response classifications from an ACT 1 sub-study. The overall study overview, evidence framework, and working model are summarized in Figure 1. Because single-probe replication was uneven across the cohorts, the downstream inference focused on recurring effect direction, convergent pathways, and sample-level scoring rather than ranking each cohort’s candidate biomarkers.

### Recurrent pretreatment inflammatory-remodeling program is associated with anti-TNF non-response

**FIGURE 2:**
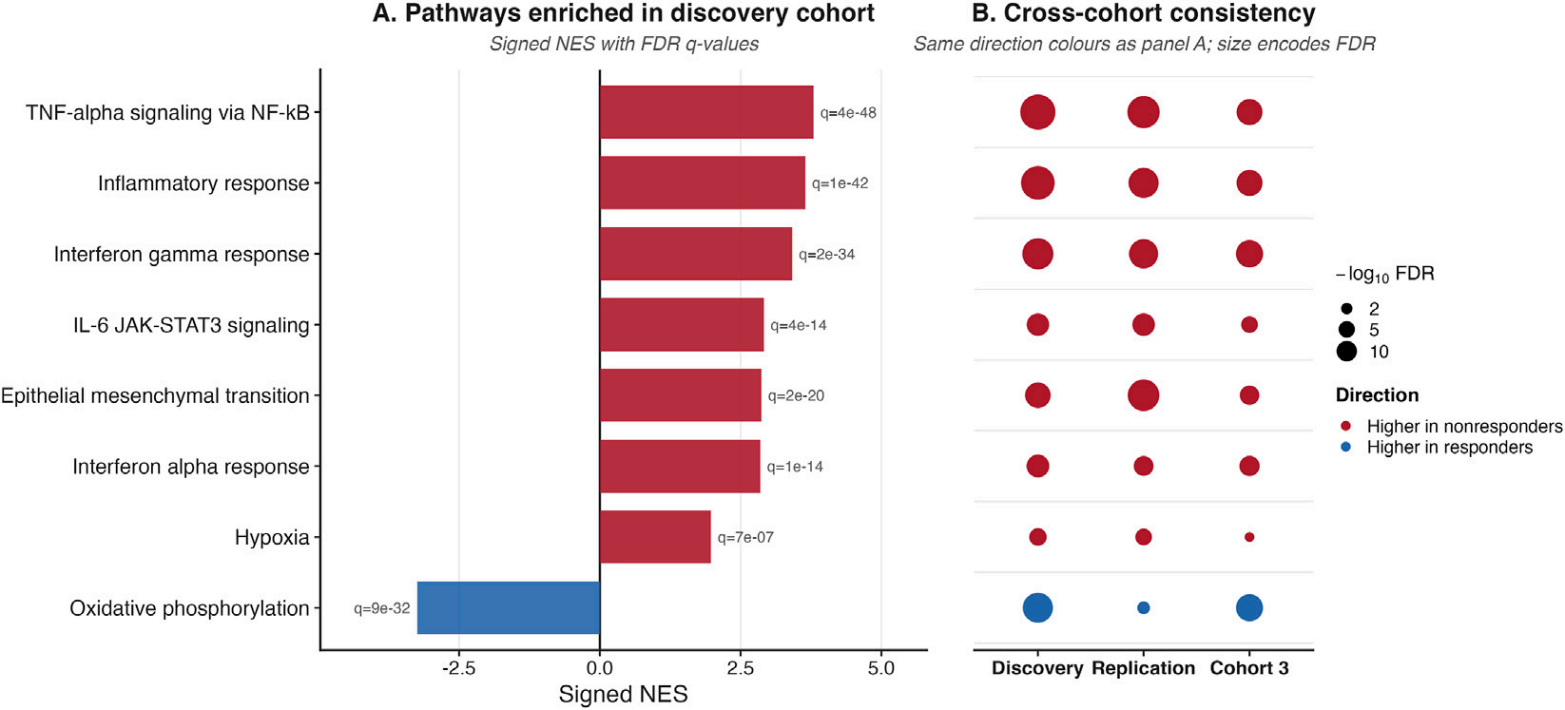
Pathway structure and cross-cohort consistency of induction-phase non-response. **(A)** Hallmark enrichment in the discovery cohort (GSE12251) is shown as signed normalized enrichment score (NES) values with false discovery rate (FDR) *q*-value labels; positive values indicate enrichment in non-responders, while negative values indicate enrichment in responders. **(B)** Cross-cohort consistency of the same pathways across GSE12251, GSE16879, and GSE23597, where the bubble size denotes FDR strength and color denotes direction based on the same non-responder/responder palette as **(A)**. The full per-cohort hallmark enrichment outputs are provided in the Supplementary Tables.

Across the cohorts, the non-responders shared a recurring inflammatory and tissue-remodeling program that included *TLR2*, *IL1B*, *IL1RN*, *PTGS2*, *TNFAIP6*, *TREM1*, *CXCR2*, *NAMPT*, *CLEC4E*, and *CD93*. Several of these genes are consistent with prior reports of mucosal inflammation and anti-TNF response biology, placing the present findings within an established biological context rather than proposing a wholly new pathway (Cui et al., 2021; Arijs et al., 2009a; Arijs et al., 2009b; Toedter et al., 2011; West et al., 2017). The pathway analyses converged on an inflammatory-remodeling program in pretreatment non-responders, including inflammatory responses, TNF-α/NF-κB signaling, IL-6–JAK–STAT3 signaling, interferon signaling, hypoxia, and EMT. In contrast, oxidative phosphorylation was reproducibly enriched in the responders. These pathway results are summarized in Figure 2.

### Consensus 68-gene signature and held-out LODO evaluation support cross-cohort association

The 68-gene consensus signature defined from the genes upregulated in the non-responders and nominally significant at *p* < 0.01 in all three cohorts was consistently higher in the pretreatment non-responders in each dataset. Because this fixed signature was derived using information from all cohorts, the within-cohort effect sizes are descriptive in-sample upper bounds (Hedges’ g = 1.85, 1.48, and 2.03 for GSE12251, GSE16879, and GSE23597, respectively), with a pooled random-effects association of Hedges’ g = 1.78 (95% CI: 1.22–2.34; *p* = 6.0 × 10^−10^; I^2^ = 0%). The signature was compositionally mixed rather than being restricted to a single canonical inflammatory marker set. Descriptive annotations of the 68 genes showed immune/myeloid and antigen-presentation components (including *CD86*, *CD93*, *FCGR2A*, *HLA-DQA1*, *LILR*-family genes, *PTPRC*, *TLR1*, and *TLR8*), inflammatory or stress-signaling mediators (including *IL-6*, *CSF3*, *PTGS2*, *SOCS3*, *IFIT3*, and *SOD2*), and remodeling or epithelial/stromal stress-associated genes (including *CHI3L1*, *COL8A1*, *HGF*, *IGFBP5*, *IL11*, *INHBA*, *SELE*, *SERPINE1*, *TFPI2*, and *ZEB1*). The full descriptive groupings are provided in Supplementary Table S1.

**FIGURE 3:**
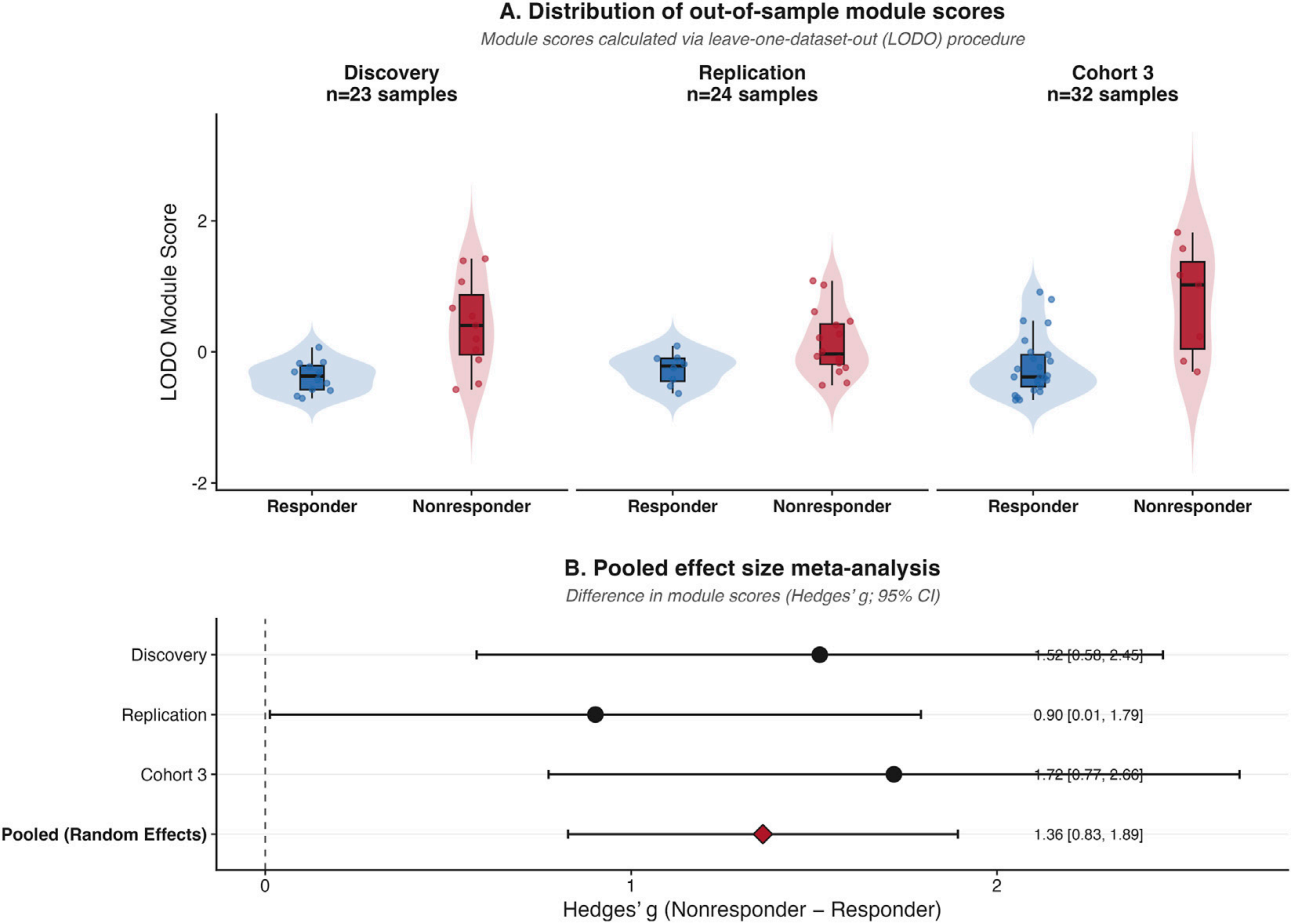
Leave-one-dataset-out (LODO) evaluation of the non-response axis. **(A)** Held-out axis scores by response status within each cohort, where each cohort is evaluated using a gene set derived from the other two cohorts. **(B)** Cohort-level standardized effect sizes (Hedges’ g; non-responder vs. responder) together with the pooled random-effects estimate.

We then investigated whether the same pattern persisted when each cohort was scored only using the genes derived from the other two cohorts. In these LODO analyses, the non-response axis remained higher in the non-responders, with effect sizes of Hedges’ g = 1.52, 0.90, and 1.72 for held-out GSE12251, GSE16879, and GSE23597, respectively. The random-effects pooling yielded Hedges’ g = 1.36 (95% CI: 0.83–1.89; *p* = 5.65 × 10^−7^; I^2^ = 0%). The LODO-derived gene sets were intentionally broader than the fixed consensus signature: 632, 1,007, and 524 genes were used when holding out GSE12251, GSE16879, and GSE23597, respectively. The 68 fixed consensus genes were contained within each LODO gene set, indicating biological consistency between the compact interpretation signature and broader training-derived held-out axes while preserving their different analytical roles. This comparison is provided in Supplementary Table S7. The LODO score distributions and pooled effect-size summaries are shown in Figure 3.

### Public single-cell references support localization to both myeloid and epithelial compartments

**FIGURE 4:**
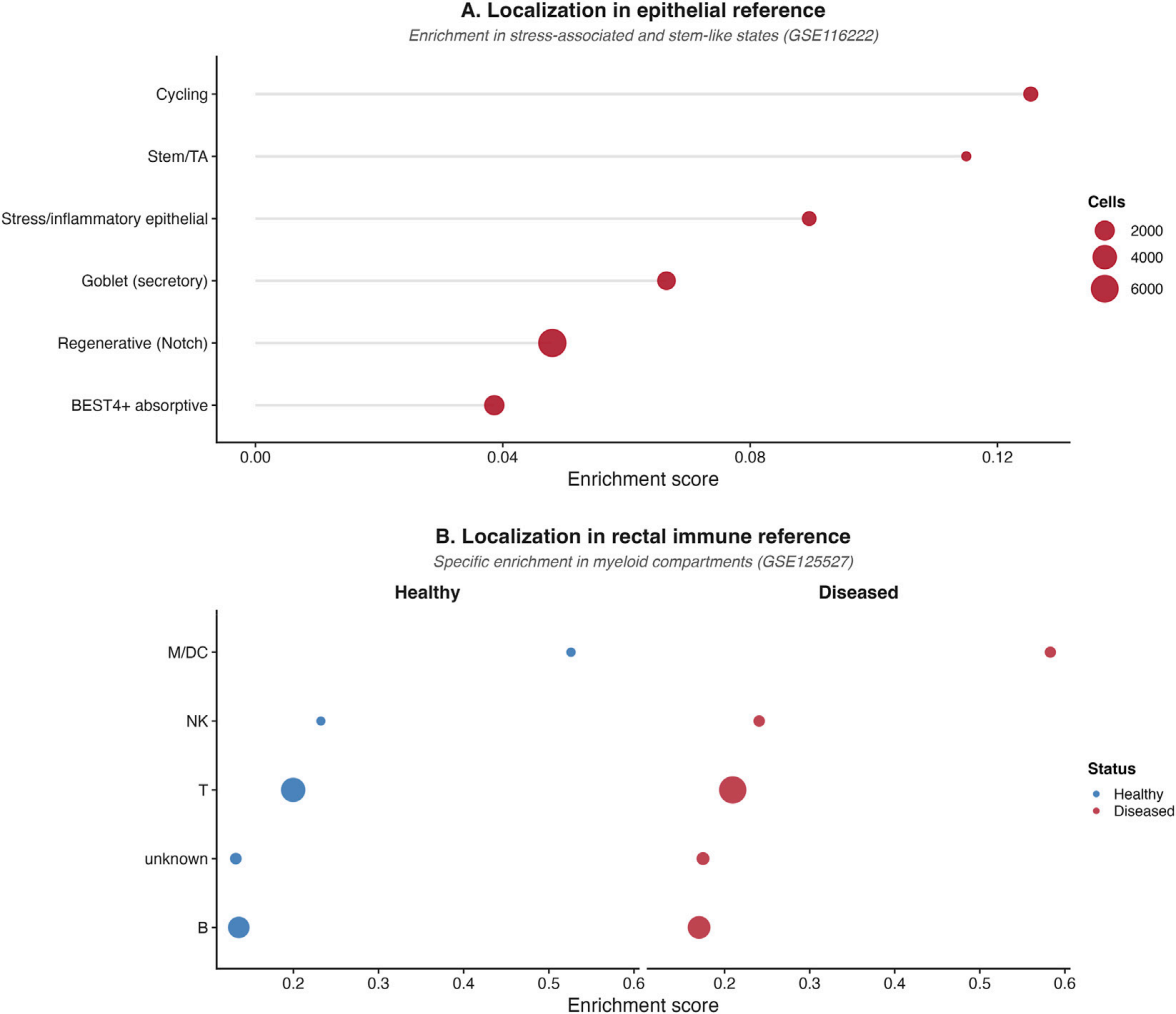
Cellular context from the processed public single-cell references. **(A)** In the epithelial reference (GSE116222), consensus-signature activity is relatively higher in the cycling/stem-like and stress-inflammatory epithelial states. **(B)** In the rectal immune reference (GSE125527), consensus-signature activity is highest in the myeloid/dendritic cells (M/DCs) relative to the lymphoid compartments. Together, these results support a mixed myeloid-inflammatory and epithelial-remodeling pattern.

Two processed public single-cell references were used to place the bulk signal within the cellular context. In the epithelial reference GSE116222, the consensus-signature activity differed across marker-defined epithelial states (Kruskal–Wallis *p* = 3.91 × 10^−219^) and was relatively higher in the cycling, stem/transit-amplifying, and stress-inflammatory epithelial states while being lower in the absorptive states (Smillie et al., 2019; Parikh et al., 2019). The one-versus-rest tests supported higher signature activities in the cycling, stem/transit-amplifying, and stress-inflammatory epithelial states after Benjamini–Hochberg adjustment (Supplementary Table S7). In the rectal immune reference GSE125527, the consensus-signature activity differed across immune cell classes in both diseased and healthy rectal cells (Kruskal–Wallis *p* < 1 × 10^−300^ for each subset). The signal was highest in the myeloid/dendritic cells (M/DCs) in diseased rectal tissue (mean score of 0.583), exceeding those of the NK (0.241), T (0.210), and B (0.170) cell compartments. The M/DCs remained higher than all other diseased rectal immune cells in the one-versus-rest tests after Benjamini–Hochberg adjustment (adjusted *p* = 1.05 × 10^−156^). The same rank order was observed in the healthy rectum, where the M/DCs again showed the highest average score (0.526) (Boland et al., 2020). Some representative consensus gene-expression patterns across the epithelial and immune cell populations are shown in Supplementary Figure S3. Together, these two references point to a mixed pattern with a prominent myeloid/inflammatory component alongside epithelial stress/proliferative activity. The single-cell localization support is summarized in Figure 4.

#### Baseline inflammatory burden explains part of the non-response axis

**FIGURE 5:**
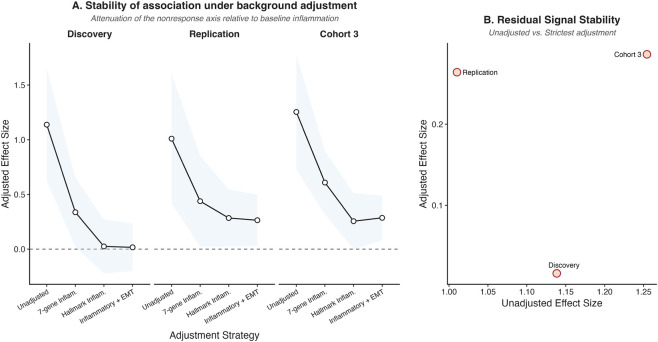
Attenuation of the consensus-signature-response association after adjustment for baseline inflammation/remodeling. **(A)** Stepwise changes in association under increasingly strict adjustments using a simple inflammatory proxy, a hallmark inflammatory proxy, and a hallmark inflammatory-plus-EMT proxy. **(B)** Comparison of the unadjusted and strictly adjusted effects across cohorts. Supplementary Figure S2 summarizes the per-sample relationships between the held-out LODO axis scores and baseline inflammatory/remodeling proxies and presents a complementary partial-residual view after proxy adjustment.

Adjustment for a simple inflammatory proxy attenuated the non-response association, but the positive residual coefficients remained across all three cohorts. With stricter hallmark inflammatory and inflammatory-plus-EMT adjustments, the response-status coefficient remained positive in all cohorts and retained nominal significance in the GSE16879 (*p* = 0.030) and GSE23597 (*p* = 0.0077) datasets under the strictest joint model. In contrast, in the discovery cohort GSE12251, the adjusted coefficient was essentially null (beta = 0.016, *p* = 0.88), indicating that the signature-response association in this small cohort is statistically collinear with the baseline mucosal inflammatory and tissue-remodeling burden. These analyses place the non-response axis close to baseline inflammatory severity while showing residual response-associated signals in the two replication cohorts after the same proxy adjustments. The specificity benchmarks are summarized in Figure 5.

As an external class-level anti-TNF sensitivity analysis, the 68-gene consensus signature was also directionally higher in week-6 non-responders in the golimumab-treated PURSUIT-SC cohort (GSE92415; pretreatment biopsies; n = 87), with a modest but significant standardized association (Hedges’ g = 0.50, 95% CI: 0.07–0.92; *p* = 0.022; Supplementary Table S6; Supplementary Figure S1) (Sandborn et al., 2014; Telesco et al., 2018).

## Discussion

### Main interpretation

This study identifies a reproducible pretreatment mucosal state associated with induction-phase non-response in infliximab-treated UC cohorts. The data support a broader inflammatory-remodeling axis that recurs across three infliximab-treated cohorts, remains evident in the held-out LODO analyses, and is most active in rectal M/DCs together with stress-associated epithelial states. The main contribution of this work is a replicated biological interpretation of pretreatment heterogeneity and not a standalone predictive test.

#### Biological context

The enrichment pattern in the rectal M/DC compartments, together with the TNF-α/NF-κB, IL-6–JAK–STAT3, interferon, and hypoxia programs, is consistent with a cytokine-rich myeloid inflammatory milieu coupled to epithelial stress and remodeling. Prior works have likewise implicated myeloid/stromal inflammatory states, including OSM-driven biology, in anti-TNF non-response in IBD (West et al., 2017; Thomas et al., 2024; Gudiño et al., 2025). To understand how the 68-gene consensus signature relates to these established frameworks, we compared its components with the classical OSM-driven axis. While the OSM axis is primarily stromal and macrophage-centric, our 68-gene signature captures the downstream transcriptional endpoints of myeloid activation (such as *CD86*, *LILRB* genes, and *CSF2RB*) alongside direct downstream targets of IL-6/OSM signaling (such as *SOCS3* and *BCL6*). This places the consensus signature as a broader downstream readout of the myeloid-remodeling axis.

The single-cell results add an epithelial layer to this interpretation. The consensus-signature activity was concentrated in both cycling/stem-like epithelial cells and M/DCs: a pattern compatible with lamina propria myeloid activation occurring alongside epithelial regeneration and stress remodeling. A plausible tissue-level model for this is that inflammatory cytokines, such as IL-6, TNF-α, and OSM, act on the epithelial progenitor and reparative compartments during active mucosal injury, producing a proliferative and stress-associated state marked by hypoxia and EMT-related programs. In the pretreatment biopsies, high activities of this myeloid-epithelial remodeling axis may therefore indicate a refractory inflammatory tissue state. Because the primary cohorts were sampled before treatment, this interpretation remains broader than anti-TNF-specific resistance alone and includes the possibility of general inflammatory refractoriness.

### Specificity and confounding

An important interpretive challenge is separating treatment resistance from the baseline inflammatory burden. In our data, adjustment for the inflammatory and EMT-related proxies reduced the response-status coefficient substantially but did not remove the association across cohorts. The near-null adjusted coefficient in GSE12251 (*p* = 0.88) suggests that, in this small discovery cohort, the signature-response association is statistically inseparable from the overall baseline inflammatory and tissue-remodeling burden. This may reflect a subset in which the refractory disease is dominated by inflammatory scaling or limited power (n = 23) to estimate the residual effects in the presence of strong collinearity. In contrast, the persistence of significant adjusted associations in the replication cohorts (GSE16879, *p* = 0.030; GSE23597, *p* = 0.0077) supports cross-cohort directional stability and suggests that the 68-gene signature captures the response-associated biology, including myeloid-stromal activation and epithelial stem-like stress responses, beyond simple scaling of mucosal inflammation.

## Limitations

The present study has clear limitations: it is retrospective, is based on public transcriptomic data, and cannot support causal inference. The outcome definitions were harmonized across the studies rather than being prospectively standardized, and the biologic-naive status, exact biopsy locations, medication-regimen details, and baseline endoscopic or histologic severity data were not uniformly available in the deposited annotations. The 68-gene consensus signature was selected through a stringent cross-cohort nominal threshold and directional agreement rule, but this rule was designed for reproducible biological interpretation rather than formal family-wise error control across all possible intersections. Moreover, we did not exhaustively evaluate alternative threshold or intersection/union rules for signature construction. All primary bulk cohorts used microarray platforms, which have more limited dynamic ranges and reduced sensitivities for low-abundance transcripts than next-generation sequencing. The specificity analyses used inflammatory and EMT proxy scores but did not include direct deconvolution or cell-type abundance covariates; thus, residual confounding by the cellular composition remains possible. The single-cell datasets provide cellular context rather than treatment-response validation, and the per-cell tests do not account for donor-level clustering. In addition, the immune reference was restricted to rectal tissue, whereas the bulk biopsy locations were not uniformly documented as rectal; hence, location-dependent cellular composition or gene-expression differences could influence the interpretation. The immune-reference analysis also used only the subset of signature genes represented in that matrix, and the golimumab cohort should be interpreted as supportive class-level evidence rather than broad anti-TNF generalization.

Moving toward clinically actionable stratification will require targeted validation rather than additional retrospective reanalyses alone. Therefore, the priority next steps include testing the 68-gene signature in prospective pretreatment biopsy cohorts with standardized response endpoints, comparing the signature with established inflammatory-response frameworks such as the OSM axis (West et al., 2017), and evaluating whether a smaller quantitative polymerase chain reaction or targeted RNA panel can reproduce the signal. Future models should also include endoscopic severity, histology, C-reactive protein, fecal calprotectin, drug exposure, and immunogenicity variables because therapeutic drug monitoring (Brun et al., 2024) and anti-drug antibody or HLA-associated immunogenicity (Culmsee-Holm et al., 2025; Zhang et al., 2025; Hodges et al., 2026) can affect apparent treatment failure independently of the baseline tissue state. Mechanistic validation should focus on the main biological components supported by the present findings: myeloid/stromal inflammatory programs (Korsunsky et al., 2022; Melón-Ardanaz et al., 2025; Wang et al., 2025b), epithelial stress and remodeling (Korsunsky et al., 2022; Melón-Ardanaz et al., 2025; Johnson et al., 2024), hypoxia/stress pathways (Hou et al., 2024; Di Mattia et al., 2024), pH-sensing inflammatory pathways (Hausmann et al., 2024), proteomic disease-activity signatures (Veyssière et al., 2025), microbial interaction networks (Melograna et al., 2024), and candidate inflammatory targets such as *FPR2* (Yang et al., 2026). Overall, induction-phase non-response in the infliximab-treated UC cohorts is associated with a reproducible pretreatment mucosal inflammatory-remodeling state that maps most strongly to the rectal M/DCs and epithelial stress/proliferative states in public single-cell references. The association is attenuated but not extinguished after accounting for the baseline inflammatory/remodeling burden.

## Data Availability

Publicly available datasets were analyzed in this study. All datasets analyzed in this study are publicly available from the NCBI Gene Expression Omnibus (GEO) database: GSE12251 (https://www.ncbi.nlm.nih.gov/geo/query/acc.cgi?acc=GSE12251), GSE16879 (https://www.ncbi.nlm.nih.gov/geo/query/acc.cgi?acc=GSE16879), GSE23597 (https://www.ncbi.nlm.nih.gov/geo/query/acc.cgi?acc=GSE23597), GSE92415 (https://www.ncbi.nlm.nih.gov/geo/query/acc.cgi?acc=GSE92415), GSE116222 (https://www.ncbi.nlm.nih.gov/geo/query/acc.cgi?acc=GSE116222), and GSE125527 (https://www.ncbi.nlm.nih.gov/geo/query/acc.cgi?acc=GSE125527). Source code and the public reproducibility package are available at GitHub (https://github.com/Zhenghongwei11/Pretreatment-nonresponse-axis-in-UC; release v1.2.1) and are archived on Zenodo (version-specific DOI: 10.5281/zenodo.21496340; concept DOI: 10.5281/zenodo.19028074).
