# Supplementary material for: A reproducible pretreatment mucosal-inflammatory-remodeling state is associated with induction-phase anti-tumor necrosis factor non-response in ulcerative colitis
Source: Front Genet. 2026 Jul 30;17:1845510. doi: 10.3389/fgene.2026.1845510 (PMC13466229; doi:10.3389/fgene.2026.1845510)
Supplement: Supplementary file 1 [file DataSheet1.pdf]

Supplementary Figures

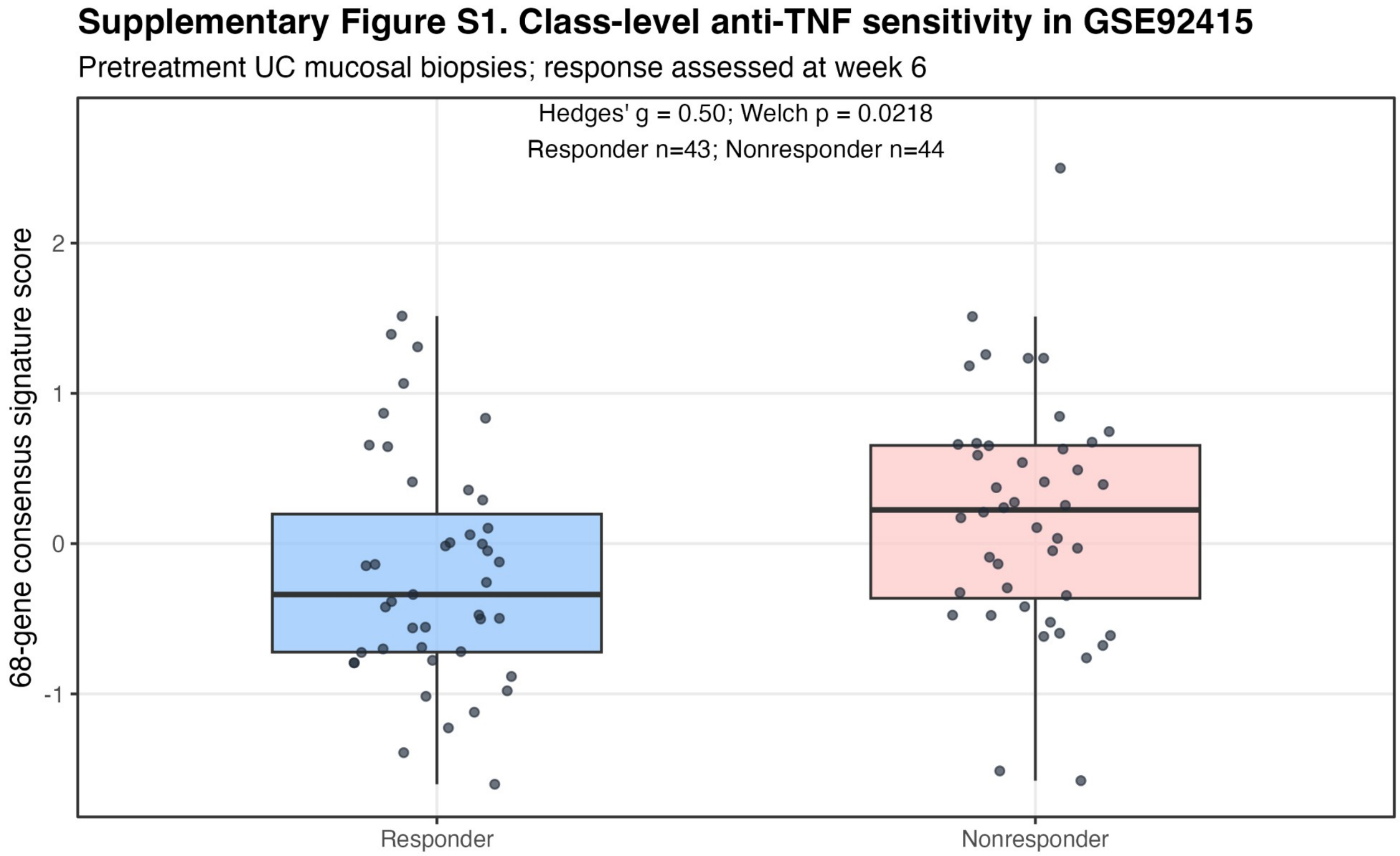

Supplementary Figure S1. Class-level anti-TNF sensitivity analysis in the golimumab-treated PURSUIT-SC cohort (GSE92415). The boxplot and jitter plot show pretreatment 68-gene consensus-signature scores by Week 6 response status (Responders, n=43; Nonresponders, n=44). Jitter points represent individual mucosal biopsy samples. The consensus signature score was directionally higher in week 6 nonresponders (Welch's t-test P = 0.022, Hedges' g = 0.50, 95% CI 0.07-0.92). Box boundaries denote the interquartile range (IQR), center lines denote the median, and whiskers extend to 1.5 times the IQR.

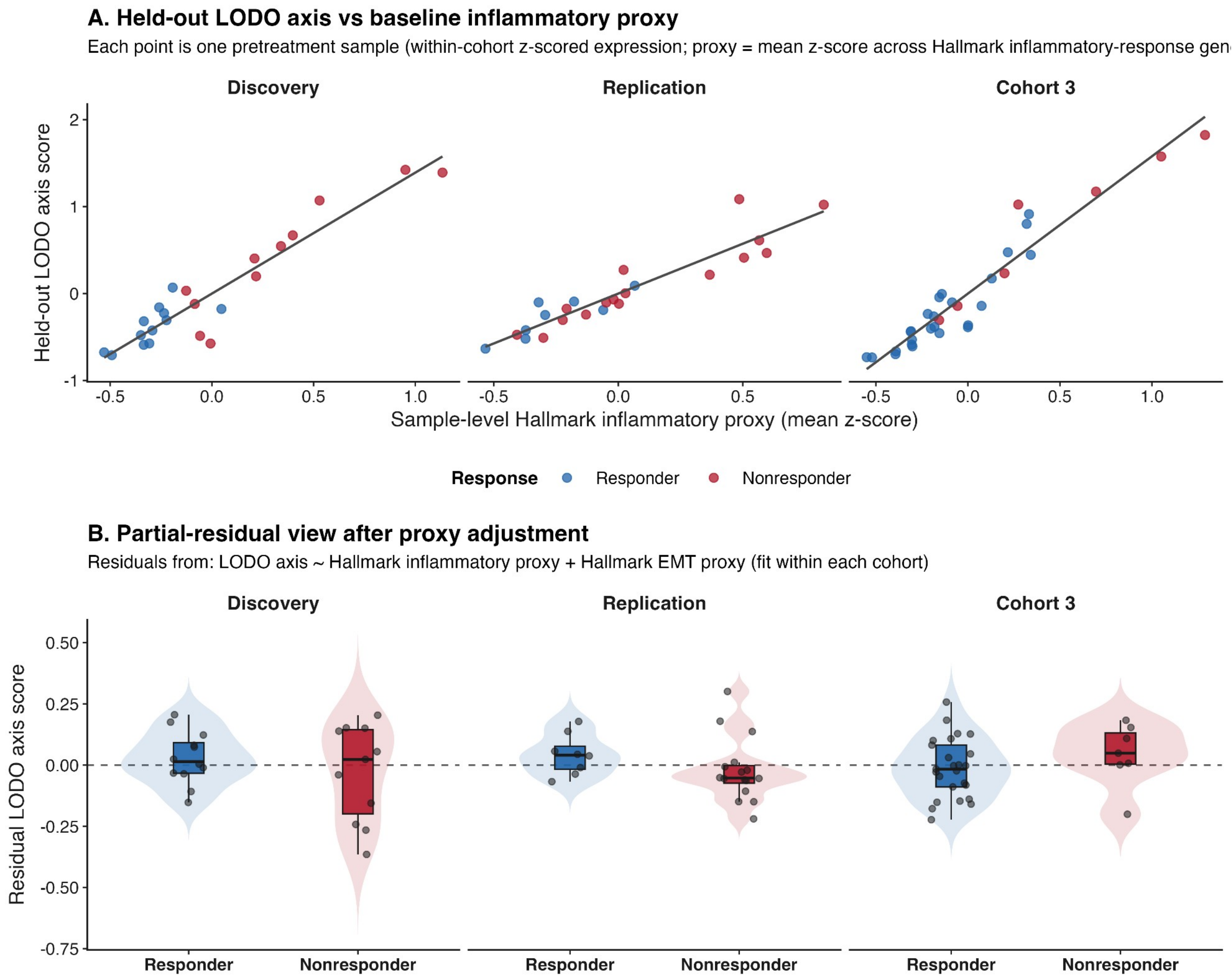

Supplementary Figure S2. Relationships between held-out LODO axis scores and baseline inflammatory/remodeling proxies across Discovery, Replication, and Cohort 3 datasets. (A) Scatter plots of pretreatment sample-level Hallmark inflammatory proxy scores vs. held-out LODO axis scores. Regression lines indicate significant baseline correlation within each cohort. (B) Boxplots and violin plots of residual LODO axis scores after strict covariate adjustment (LODO score adjusted for Hallmark inflammatory and EMT proxies fit within each cohort) in Responders vs. Nonresponders. The horizontal dashed line indicates zero residual.

Supplementary Figure S3. Expression pattern of representative consensus-signature genes

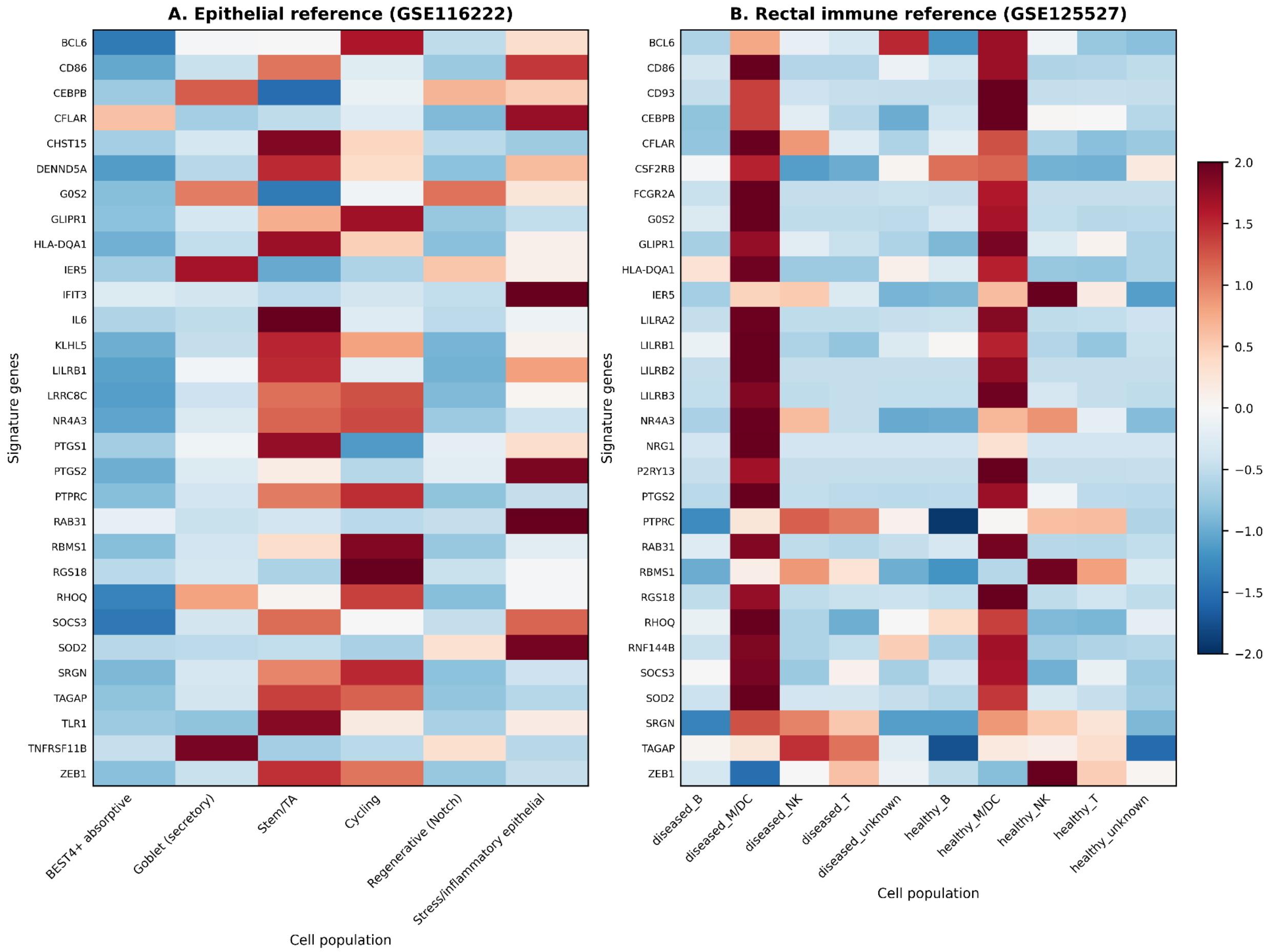

Supplementary Figure S3. Heatmaps of representative consensus-signature gene expression across single-cell reference populations. Row z-scores show mean log-normalized expression of the most variable available consensus-signature genes across epithelial states in GSE116222 and rectal immune cell classes in GSE125527.
